# Esophageal submucosal tumor diagnosed with EBUS‐guided transbronchial mediastinal cryobiopsy: A case report

**DOI:** 10.1111/1759-7714.14650

**Published:** 2022-09-13

**Authors:** Yuki Ishiguro, Keigo Uchimura, Hideaki Furuse, Tatsuya Imabayashi, Yuji Matsumoto, Shun‐Ichi Watanabe, Takaaki Tsuchida

**Affiliations:** ^1^ Department of Endoscopy, Respiratory Endoscopy Division National Cancer Center Hospital Tokyo Japan; ^2^ Department of Thoracic Surgery National Cancer Center Hospital Tokyo Japan; ^3^ Department of Thoracic Oncology National Cancer Center Hospital Tokyo Japan

**Keywords:** bronchoscopy, cryobiopsy, endobronchial ultrasound‐guided transbronchial needle aspiration, esophageal neoplasms, leiomyoma

## Abstract

Cryobiopsy is advantageous for collecting larger specimens with minimum crushing compared to forceps biopsy and transbronchial needle aspiration (TBNA), but it has not been widely used for mediastinal tumors. In this report, a leiomyoma of the thoracic esophagus was diagnosed with endobronchial ultrasound‐guided transbronchial mediastinal cryobiopsy (EBUS‐cryo). An asymptomatic 49‐year‐old woman had a 2.6‐cm sized submucosal tumor (SMT) of the esophagus adjacent to the trachea and left main bronchus. EBUS‐TBNA and EBUS‐guided intranodal forceps biopsy were performed, followed by EBUS‐cryo. The biopsy forceps could not be inserted into the tumor, but the cryoprobe was smoothly inserted. EBUS‐TBNA could not obtain enough spindle‐shaped tumor cells for immunohistochemical staining, but EBUS‐cryo provided sufficient specimens for diagnosing the leiomyoma. Adding EBUS‐cryo to EBUS‐TBNA has recently been reported to achieve high diagnostic yields for lymphomas, uncommon tumors, and benign diseases. EBUS‐cryo seems a valid diagnostic option for esophageal SMTs that are difficult to diagnose with needles and forceps.

## Graphical abstract

We present a case of leiomyoma of the thoracic esophagus diagnosed with endobronchial ultrasound‐guided transbronchial mediastinal cryobiopsy (EBUS‐cryo). Unlike endobronchial ultrasound‐guided transbronchial needle aspiration, EBUS‐cryo provided sufficient specimens for a leiomyoma diagnosis without serious complications. EBUS‐cryo is a valid option for esophageal submucosal tumors that are difficult to diagnose with needle and forceps biopsy.

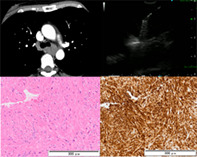

## INTRODUCTION

Endobronchial ultrasound‐guided transbronchial needle aspiration (EBUS‐TBNA) is a standard sampling technique widely used for benign and malignant mediastinal and hilar lesions.^1^, ^2^ Although its sensitivity and specificity are high for staging non‐small‐cell lung cancer, diagnostic yields for non‐cancer diseases are insufficient.^1^, ^2^, ^3^, ^4^, ^5^, ^6^ Recent studies have shown that adding EBUS‐guided intranodal forceps biopsy (EBUS‐IFB) or EBUS‐guided transbronchial mediastinal cryobiopsy (EBUS‐cryo) to EBUS‐TBNA provides higher diagnostic yields for lymphoma, uncommon tumors, and benign diseases compared to EBUS‐TBNA alone.^5^, ^6^, ^7^, ^8^, ^9^

Endoscopic ultrasound‐guided fine needle aspiration (EUS‐FNA), which is also a needle‐based technique, is commonly used to diagnose gastrointestinal submucosal tumors (SMTs).^10^, ^11^ The diagnosis of SMTs requires tissue specimens that can be evaluated immunohistochemically, but limited EUS‐FNA specimens are suitable for immunohistochemical staining.^12^, ^13^, ^14^ Cryobiopsy is advantageous for collecting larger specimens with minimum crushing compared to forceps biopsy and TBNA.^15^, ^16^, ^17^ A recent randomized trial reported that EBUS‐cryo was as safe as EBUS‐TBNA,^7^ therefore EBUS‐cryo might be a breakthrough technique in the diagnosis of SMTs.

Here, we report a case of SMT of the thoracic esophagus diagnosed with EBUS‐cryo without serious complications.

## CASE REPORT

**FIGURE 1:**
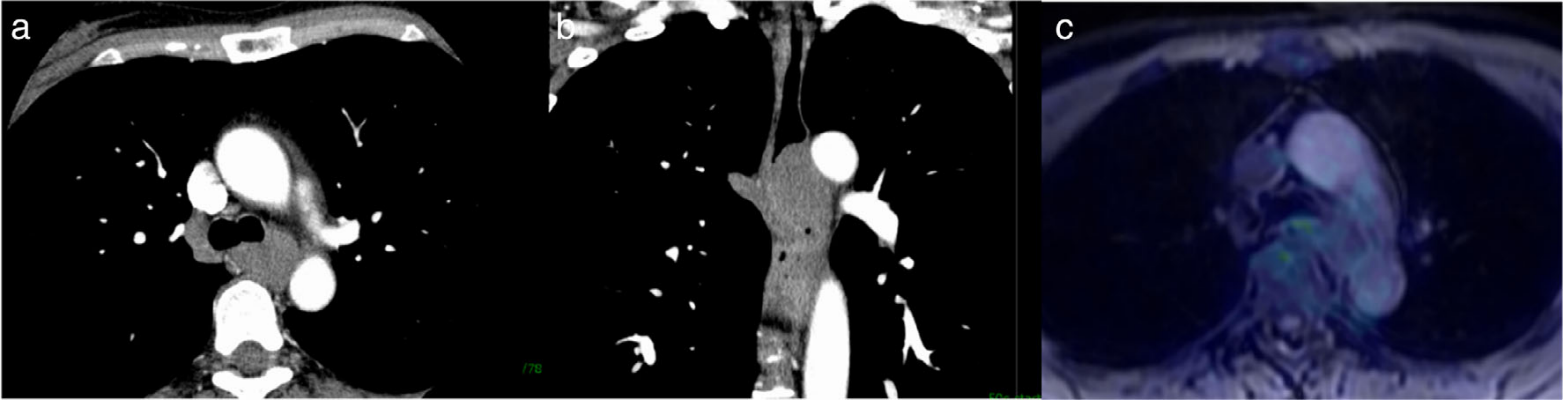
Chest computed tomography (CT) and positron emission tomography (PET)–magnetic resonance imaging (MRI). (a, b) Chest CT shows a 2.6‐cm‐sized tumor adjacent to the left main bronchus and trachea, which was contiguous with the thoracic esophagus (a, axial image; b, coronal image). (c) The tumor showed fluorodeoxyglucose uptake with a maximum standardized uptake value of 2.19 on PET‐MRI (c, axial image)

**FIGURE 2:**
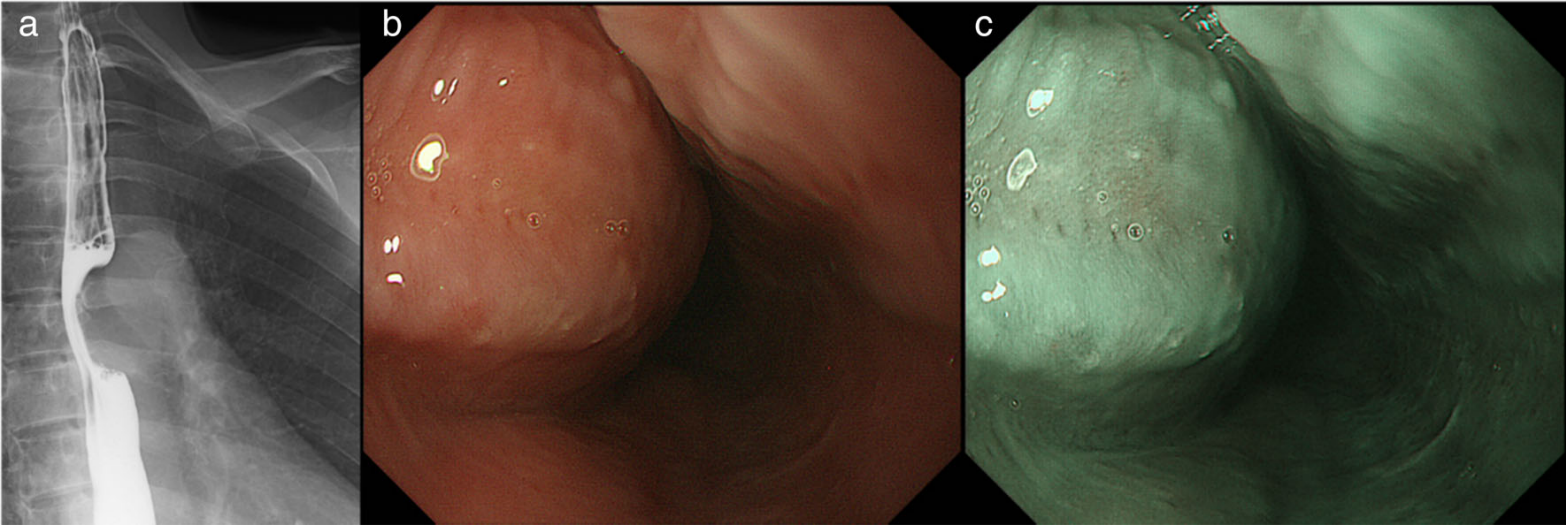
Upper gastrography and upper gastrointestinal endoscopy. (a) Upper gastrography showed a tumor with extramural compression of the upper/middle thoracic esophagus. (b, c) Upper gastrointestinal endoscopy showed extramural compression covered by normal mucosa, which was indicative of a submucosal tumor in the upper/middle thoracic esophagus (b, white‐light image; c, narrow‐band image)

A 49‐year‐old woman was referred for the diagnosis of a mediastinal tumor. She was asymptomatic and without a previous history of tumor. Chest computed tomography showed a 2.6‐cm sized tumor adjacent to the trachea and left main bronchus, which was contiguous with the thoracic esophagus (Figure 1a,b) and did not demonstrate fluorodeoxyglucose uptake on positron emission tomography–magnetic resonance imaging (Figure 1c). Upper gastrography and gastrointestinal endoscopy revealed extramural compression covered by normal mucosa in the upper/middle thoracic esophagus (Figure 2a–c).

**FIGURE 3:**
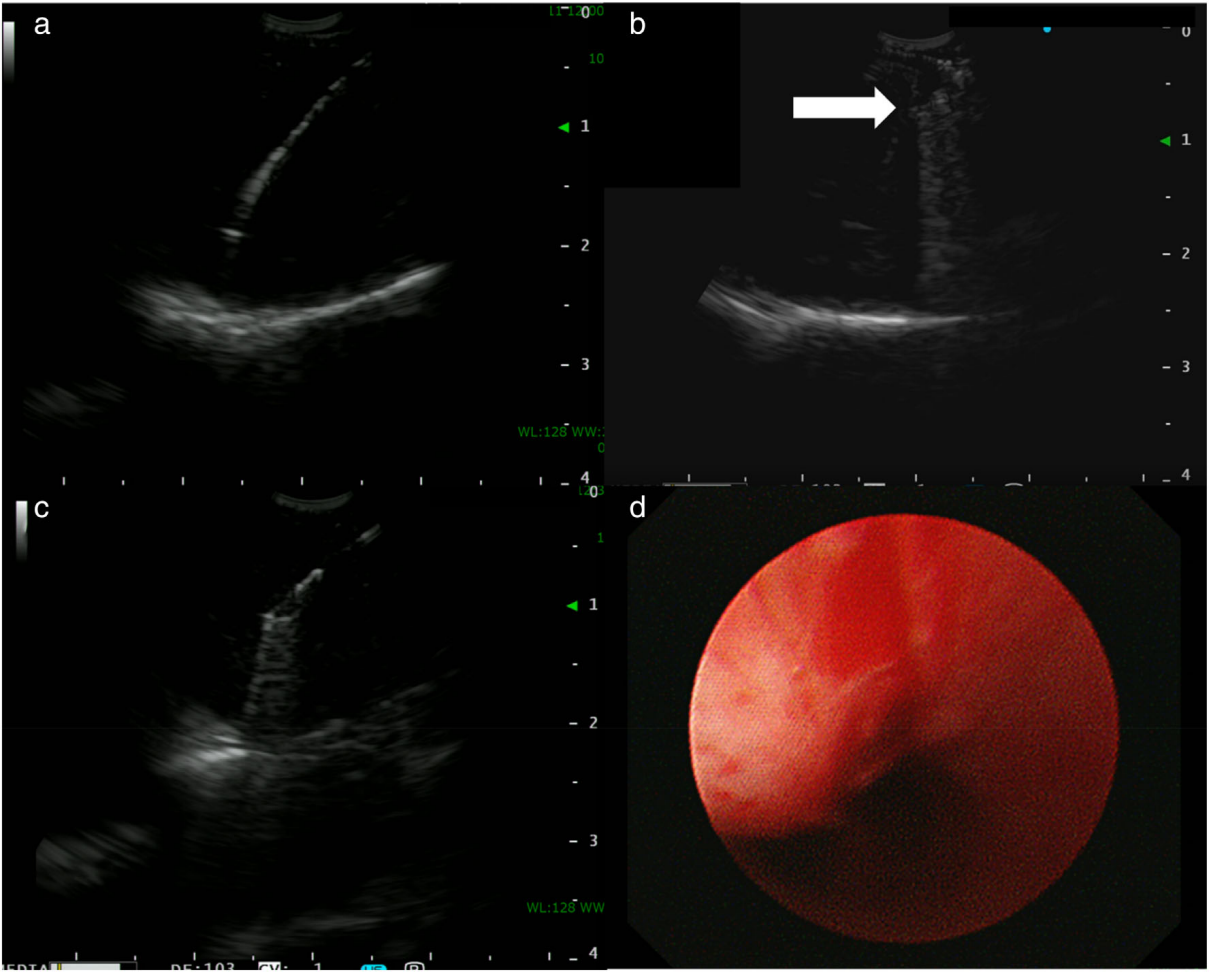
Endobronchial ultrasound (EBUS) images during bronchoscopy and post‐cryobiopsy bronchoscopic findings. (a) An EBUS image during EBUS‐guided transbronchial needle aspiration (TBNA) for a submucosal tumor of the esophagus. (b) An EBUS image during EBUS‐guided intranodal forceps biopsy for the tumor. The tip of the biopsy forceps (white arrow) passed through the airway wall through the tract formed by EBUS‐TBNA but was unable to pierce the tumor due to the tough tumor capsule. (c) An EBUS image during EBUS‐guided transbronchial mediastinal cryobiopsy for the tumor. The cryoprobe was smoothly inserted into the tumor beyond the tumor capsule through the tract formed by EBUS‐TBNA. (d) Post‐cryobiopsy bronchoscopic findings of a tract formed in the airway wall and slight temporary bleeding in the absence of any complications requiring intervention

For diagnosis, EBUS‐TBNA, comprising three punctures with a 22‐gauge needle (EchoTip Ultra; Cook Medical) and a convex probe ultrasound bronchoscope (BF‐UC290F; Olympus), was performed (Figure 3a), followed by an attempt at EBUS‐IFB with biopsy forceps (FB‐15C1; Olympus). The forceps passed the tracheal wall through the tract formed by EBUS‐TBNA but could not penetrate the tough tumor capsule (Figure 3b and Video S1), therefore we performed EBUS‐cryo with a cryoprobe (20402‐410; ERBE). The cryoprobe was smoothly inserted into the tumor (Figure 3c and Video S1), and two additional cryobiopsies were performed. The patient had a temporary cough without other complications (Figure 3d).

**FIGURE 4:**
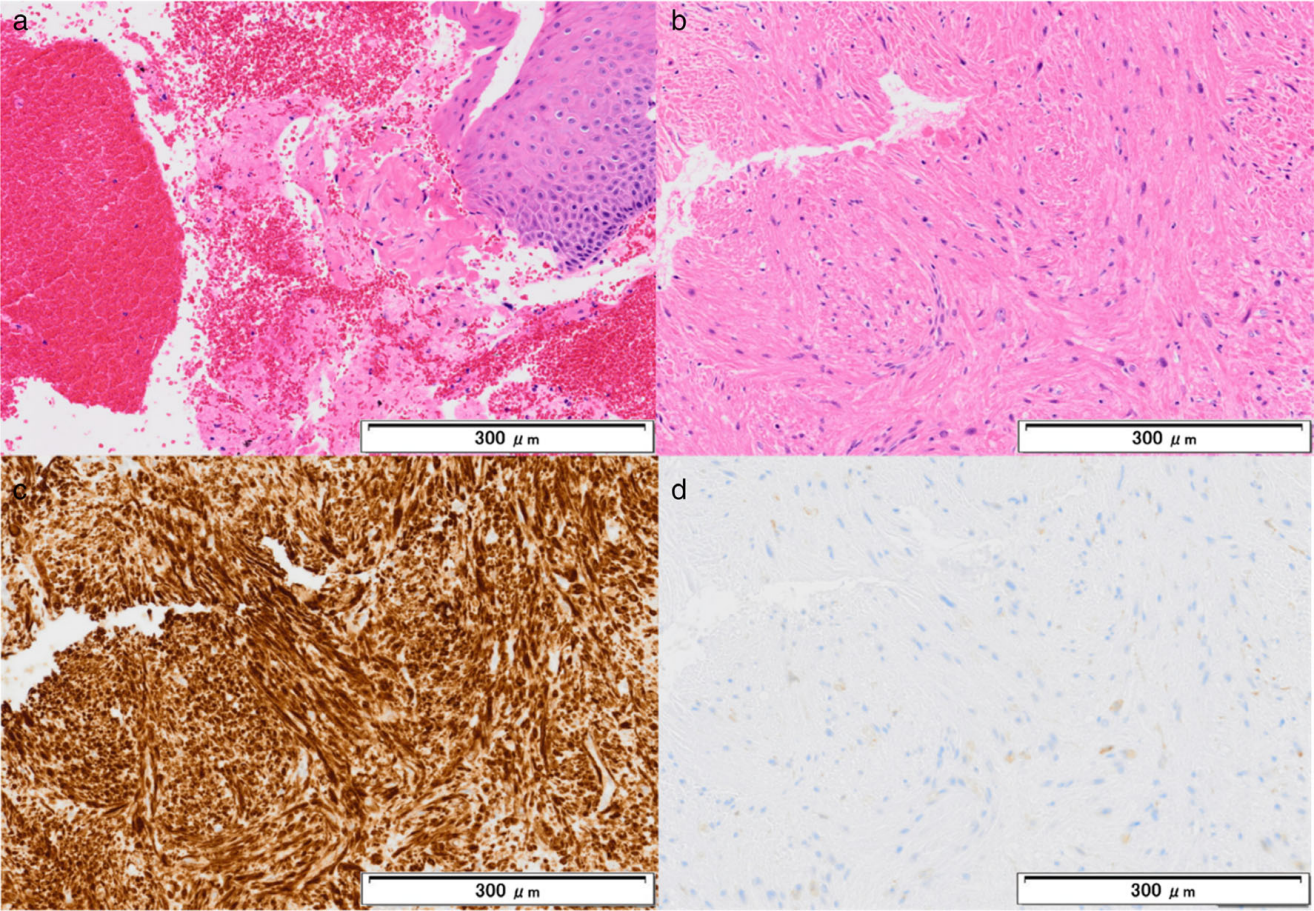
Pathological findings of the specimens obtained via bronchoscopy. (a) Endobronchial ultrasound‐guided transbronchial needle aspiration specimens showed some spindle‐shaped cells with blood and cartilage contamination, but the specimens were insufficient for diagnostic immunohistochemical staining (a, hematoxylin and eosin staining). (b–d) Cryobiopsy specimens showed proliferation of spindle‐shaped tumor cells, arranged in intersecting fascicles (b), appearing diffusely positive for desmin (c), but negative for c‐kit (d), leading to the diagnosis of leiomyoma (b, hematoxylin and eosin staining; c, desmin staining; d, c‐kit staining)

Pathologically, EBUS‐TBNA specimens showed some spindle‐shaped cells, but they were insufficient for diagnostic immunostaining (Figure 4a). The cryobiopsy specimens showed proliferation of spindle‐shaped tumor cells arranged in intersecting fascicles, appearing diffusely positive for desmin but negative for c‐kit and DOG‐1 on immunohistochemical examination (Figure 4b–d). This led to a diagnosis of leiomyoma. The tumor was observed for a year without progression.

## DISCUSSION

We presented a case of esophageal SMT diagnosed with EBUS‐cryo. EBUS‐TBNA has high sensitivity of 88–93% for staging non‐small‐cell lung cancer.^3^, ^4^ However, needle‐based specimens such as EBUS‐TBNA and EUS‐FNA are considered inadequate for immunostaining in many cases due to tissue volume, blood contamination, and crushing.^5^, ^6^, ^12^, ^13^, ^14^ The addition of EBUS‐IFB or EBUS‐cryo to EBUS‐TBNA has been reported to have diagnostic utility, especially for lymphomas, uncommon tumors, and benign diseases.^5^, ^6^, ^7^, ^8^, ^9^ A prospective study reported that only 46% of EUS‐FNA specimens for SMT were immunostainable,^13^ and the diagnostic yield for SMTs was insufficient, ranging from 46% to 84%,^12^, ^13^, ^14^ therefore biopsy methods for SMTs with high diagnostic rates need to be established.

Cryobiopsy is widely used in transbronchoscopy and is advantageous for collecting larger specimens, with minimum crushing, compared to forceps biopsy and TBNA.^15^, ^16^, ^17^ Although no studies have directly compared the diagnostic yields between EBUS‐IFB and EBUS‐cryo, EBUS‐cryo may be superior to EBUS‐IFB in terms of specimen quality and quantity. A randomized trial of EBUS‐cryo among 197 patients reported two cases of pneumothorax (1.0%) and one case of pneumomediastinum (0.5%), without serious complications.^7^ Moreover, although the safety of transesophageal cryobiopsy has not been established, a case of mediastinal tumor diagnosed as Hodgkin's lymphoma via endoscopic transesophageal cryobiopsy without complications has recently been reported.^18^ To our knowledge, a transbronchial approach for esophageal SMTs has not yet been reported, but cryobiopsy, either transbronchial or transesophageal, may be a breakthrough modality for diagnosing SMTs that need to be differentiated from gastrointestinal stromal tumors and other tumors for treatment selection.

One disadvantage of EBUS‐cryo and EBUS‐IFB techniques is that the devices can be obstructed by the airway wall and tumor capsule during insertion into the target lesion via the tract formed by TBNA. Previous studies have reported failure rates of 10–28% in EBUS‐IFB.^19^, ^20^ In the current case, forceps insertion was infeasible, but cryoprobe insertion was possible. We attribute this difference to the punctuality caused by the rigidity of devices that can apply tension against the tumor capsule. Although we used devices of approximately the same thickness, the cryoprobe was stiffer than the forceps. Despite differences in cryoprobe thickness and the method of tract creation, a randomized trial of EBUS‐cryo reported all of the 196 patients could be successes except for one, who could not tolerate the TBNA procedure,^7^ suggesting that the rigidity of the cryoprobe may be advantageous for its insertion into the lesion.

In conclusion, we reported a case of esophageal SMT diagnosed with EBUS‐cryo. EBUS‐cryo can be an option for diagnosing esophageal SMTs that are difficult to diagnose with needle‐based procedures.

## AUTHOR CONTRIBUTIONS

Yuki Ishiguro drafted the manuscript. Keigo Uchimura, Hideaki Furuse, Tatsuya Imabayashi, Yuji Matsumoto, Shun‐Ichi Watanabe, and Takaaki Tsuchida were in charge of the patient. Keigo Uchimura, Hideaki Furuse, Tatsuya Imabayashi, Yuji Matsumoto, Shun‐Ichi Watanabe, and Takaaki Tsuchida helped to draft the manuscript. All authors read and approved the final manuscript.

## CONFLICT OF INTEREST

The authors declare that they have no competing interests.

## Supporting information


**Video S1** Endobronchial ultrasound (EBUS) videos during EBUS‐guided intranodal forceps biopsy and EBUS‐guided transbronchial mediastinal cryobiopsy
